# Long-term effects of a cluster randomized controlled kindergarten-based intervention trial on vegetable intake among Norwegian 3–5-year-olds: the BRA-study

**DOI:** 10.1186/s13104-020-4892-x

**Published:** 2020-01-14

**Authors:** Anne Lene Kristiansen, Anine Christine Medin, Mona Bjelland, Anne Himberg-Sundet, Nanna Lien, René Holst, Lene Frost Andersen

**Affiliations:** 1 Department of Nutrition, Institute of Basic Medical Sciences, University of Oslo, PO Box 1046, Blindern, 0317 Oslo, Norway; 2 Department of Public Health, Sport and Nutrition, Faculty of Health and Sport Sciences, University of Agder, PO Box 422, 4604 Kristiansand, Norway; 3 Department of Biostatistics, Institute of Basic Medical Sciences, University of Oslo, PO Box 1122, Blindern, 0317 Oslo, Norway

**Keywords:** Preschool children, Kindergarten-based intervention, Vegetables, Long-term intervention effect, Norway, RCT

## Abstract

**Objective:**

To report on long-term effects of a cluster randomized controlled kindergarten-based intervention trial, which aimed to increase vegetable intake among Norwegian preschool children (3–5 years at baseline). The effects of the intervention at follow-up 1 (immediately post-intervention) have previously been published. This paper presents the effects of the intervention from baseline to follow-up 2 (12 months post-intervention).

**Results:**

Parental consents were obtained for 633 out of 1631 eligible children (response rate 38.8%). The effects of the intervention from baseline to follow-up 2 were assessed by mixed-model analyses taking the clustering effect of kindergartens into account. Children’s vegetable intake was reported by the parents at baseline (spring 2015), at follow-up 1 (spring 2016) and at follow-up 2 (spring 2017). No significant long-term effects in child vegetable intake were found. A mean difference of − 0.1 times per day (95% CI − 0.5, 0.2) (P = 0.44) was found for the daily frequency of vegetable intake. A mean difference of – 0.2 different kinds of vegetables eaten over a month (95% CI − 1.0, 0.7) (P = 0.70) was found and for daily amount of vegetables a mean difference of − 15.0 g vegetables (95% CI − 38.0, 8.0) (P = 0.19) was found.

*Trial registration* International Standard Randomised Controlled Trials ISRCTN51962956 (http://www.isrctn.com/ISRCTN51962956). Registered 21 June 2016 (retrospectively registered)

## Introduction

A sufficient intake of fruit and vegetable is associated with a reduced future risk of several non-communicable diseases (NCDs) [1–4]. Dietary habits are shaped in early childhood; hence, interventions to increase fruit and vegetable intake in this period of life may reduce the future risk of NCDs [5]. Research indicates that multi-component interventions might have a positive effect on fruit and vegetable intake among school-aged children [6, 7]. However, knowledge on how to successfully increase fruit and vegetable intake among preschool children is limited [5, 8–10], and only a few multi-component intervention studies among children of this age have been conducted [5]. Moreover, there is a lack of studies assessing the long-term effects (i.e. 12 months or more post-intervention) of interventions aiming to increase fruit and vegetable intake among preschool children [5, 9].

The objective of the present study was to report on long-term effects (i.e. 12 months or more post-intervention) of a cluster randomized controlled kindergarten-based intervention trial on vegetable intake among Norwegian preschool children (3–5 years at baseline). Effects of the intervention trial from baseline (spring 2015) to follow-up 1 (spring 2016) has previously been described [11], while this manuscript presents the effects of the intervention trial from baseline to follow-up 2 (spring 2017).

## Main text

### Methods

Study design and subjects have been presented earlier [12]. In brief, the BRA-study had an overall aim to improve vegetable intake (primary outcome) among preschool children through changing the food environment and food-related practices in the kindergarten and the home (secondary outcomes). Specifically, the goal of the intervention was to increase the daily frequency, monthly variety and daily amount of vegetable intake. Parents of children born in 2010–2011 in 73 public and private kindergartens in Norway (response rate 15.2%) were invited to participate. Parental consent was obtained for 633 children (response rate 38.8%). This study was conducted according to the guidelines laid down in the Declaration of Helsinki, and the Norwegian Center for Research Data approved all procedures. Data Protection Officer at the University of Oslo assessed data privacy issues according to General Data Protection Regulations (GDPR).

Multiple intervention components were implemented from September 2015 to February 2016. The design of the intervention and intervention components have been described elsewhere [11].

#### Data collection

Recordings of children’s vegetable intake was collected at baseline (n = 633), at follow-up 1 (n = 596) and at follow-up 2 (n = 567) by parental web-based questionnaires assessing frequency and variety (questionnaire Q1) and amount (questionnaire Q2) of vegetable intake. The questionnaires have been presented previously [12].

#### Data analysis

Data from baseline, follow-up 1 and follow-up 2 are included in this paper. Two-level mixed effect models for continuous outcomes were used to examine potential long-term effects of the intervention upon the children’s vegetable intake, with kindergartens and participants as random effects. Vegetable intake, specified by frequency (times/day), variety (kinds/month) and amount (g/day), were used as dependent variables in separate models. Group (control or intervention) and time (baseline, follow-up 1 and follow-up 2) were used as fixed effects. The intervention effects were defined as the differences in the change in vegetable intake between the control group and the intervention group from baseline to each of the follow-up times. These were quantified by interaction terms between group and time and tested for significance by Wald’s tests. The following covariates were adjusted for in the models: maternal educational level, age of participant, gender of participant, owner structure of kindergarten (public or private), and if the child attended school or not at follow-up 2. Participants were included in the analyses if they had data on baseline and/or follow-up 1 and/or follow-up 2 and if they had data on all covariates.

Children born in 2010 started school in August 2016, prior to follow-up 2, whereas children born in 2011 were still in kindergarten at follow-up 2. Hence, an interaction term between group and school attendance was explored. All analyses were conducted using Stata/SE 15.

### Results

From baseline to follow-up 2, 24 children (8%) were lost to follow-up in the control group and 42 children (13%) were lost to follow-up in the intervention group. Main reasons were children moving to other kindergartens and kindergartens withdrawing from the study after follow-up 1. Four kindergartens withdrew, resulting in a loss of 25 children. Drop-out analysis showed no differences in background characteristics between participating children (*n* = 633) and those lost to follow-up (*n* = 66), except that significantly more children were lost in the intervention group compared to the control group (P = 0.02) (data not shown). The interaction term between group and school attendance was not significant.

Seventy-three percent of the participants were included in analysis of the effect of the intervention from baseline to follow-up 2 (Table 1). In addition to those lost to follow-up, about half were lost due to not having dietary data at any time-point while another half was lost due to missing on covariates. No significant long-term effects in child vegetable intake were found (Table 2). A mean difference of − 0.1 times per day (95% CI − 0.5, 0.2) (P = 0.44) was found for the daily frequency of vegetable intake. A mean difference of − 0.2 different kinds of vegetables eaten over a month (95% CI − 1.0, 0.7) (P = 0.70) was found and for daily amount of vegetables a mean difference of − 15.0 g vegetables (95% CI − 38.0, 8.0) (P = 0.19) was found.

**Table 1:** Participants in the BRA-study with data on vegetable intake in the data collections

Data from Q1—frequency and variety of vegetable intake		Data from Q2—amount of vegetables consumed	
Participants with data on all covariates and data from Q1 on at least one time point *n* 462 (73%):		Participants with data on all covariates and data from Q2 on at least one time point *n* 459 (73%):	
	*n*		*n*
Baseline data only	139	Baseline data only	139
Baseline and follow-up 1 data	92	Baseline and follow-up 1 data	85
Baseline, follow-up 1 and follow-up 2 data	135	Baseline, follow-up 1 and follow-up 2 data	159
Baseline and follow-up 2 data	29	Baseline and follow-up 2 data	40
Follow-up 1 data only	28	Follow-up 1 data only	10
Follow-up 1 and follow-up 2 data	24	Follow-up 1 and follow-up 2 data	14
Follow-up 2 data only	15	Follow-up 2 data only	12

**Table 2:** Intervention effects of the BRA-study on vegetable outcome from baseline to follow-up 2

	Intervention effect*	
	Baseline to follow-up 2	
	Estimate (95% CI)	P
Frequency of vegetable intake (times/day)^a^	− 0.1 (− 0.5, 0.2)	0.44
Variety in vegetable intake (kinds/month)^a^	− 0.2 (− 1.0, 0.7)	0.70
Amount of vegetables consumed (g/day)^b^	− 15.0 (− 38.0, 8.0)	0.19

* Fixed effects parameter estimates. Adjusted for kindergarten clustering, time, child gender, child year of birth, maternal education and kindergarten ownership (private or public) and if the child attended school or not

^a^Frequency and variety of vegetable intake was calculated based on data from Q1. Participants were included in the analyses if they had data on baseline and/or follow-up 1 and/or follow-up 2 and all covariates, n = 462 (n = 236 in the control group and n = 226 in the intervention group)

^b^Amount of vegetables consumed in one day was calculated based on data from Q2. Participants were included in the analyses if they had data on baseline and/or follow-up 1 and/or follow-up 2 and all covariates, n = 459 (n = 233 in the control group and n = 226 in the intervention group)

**Fig. 1:**
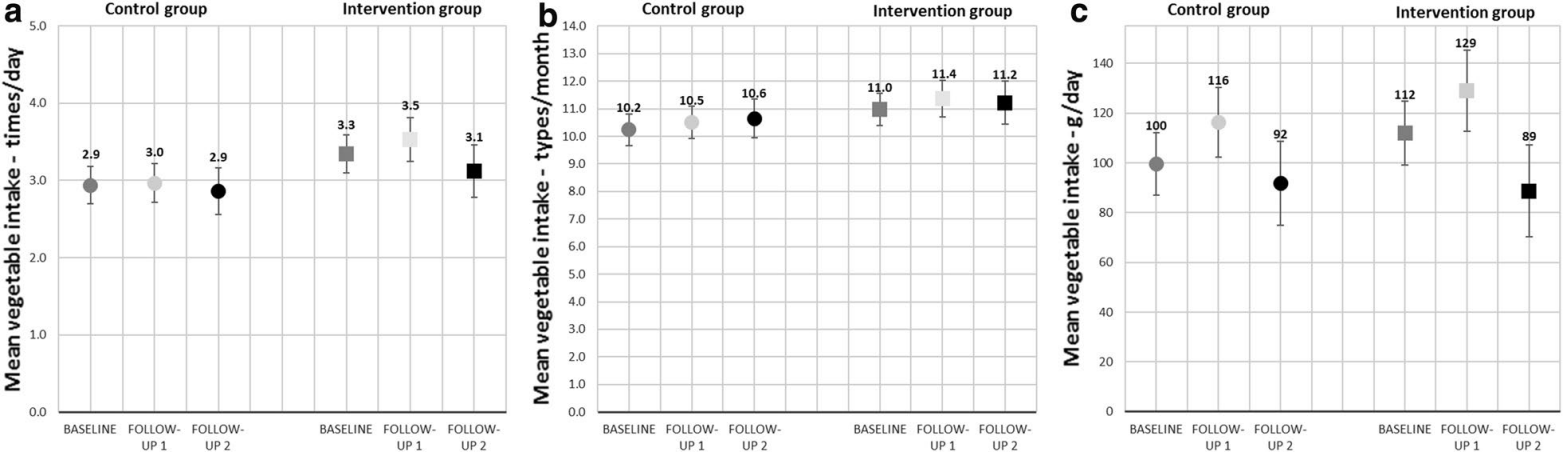
Estimated marginal means of the BRA-study, showing vegetable intake at baseline, follow-up 1 and follow-up 2, adjusted for kindergarten clustering, time, age and gender of the child, maternal education level and kindergarten ownership (private or public) and if the child attended school or not. **a** Frequency (times/day) of vegetable intake was calculated based on data from Q1, *n* 462. **b** Variety (kinds/month) of vegetable intake was calculated based on data from Q1, *n* 462. **c** Amount (g/day) of vegetables was calculated based on data from Q2, *n* 459. *○*Control group *□*Intervention group

Figure 1 shows vegetable intake in the intervention and the control group at the three time points. For frequency of vegetables, a non-significant increase from baseline to follow-up 1 was seen in both groups (Fig. 1a). From baseline to follow-up 2, no change was observed in the control group, while a small non-significant decrease was seen in the intervention group (Fig. 1a). For variety of vegetables, a non-significant increase from baseline to follow-up 1, and from baseline to follow-up 2, was seen in both groups (Fig. 1b). Daily amount of vegetables showed a significant increase from baseline to follow-up 1 by 17 g in both the intervention (P = 0.04) and the control group (P = 0.02) (Fig. 1c). From baseline to follow-up 2, a significant decrease by 23 g vegetables was seen in the intervention group (P = 0.02), whereas a non-significant decrease of 8 g vegetables was observed for the control group (P = 0.40) (Fig. 1c).

### Discussion

Actions targeting an increased vegetable intake in early childhood have been called for [5, 9], and this study aimed to contribute to fill gaps in this field with its multi-component and long-term design. However, no significant long-term effects in child vegetable intake as measured by the parental reported frequency, variety and amount of vegetables were found.

In contrast to findings in the present study, which was entirely based on self-reported dietary data, a borderline significant increase in amount of vegetables in favor of the intervention group was found at follow-up 1, assessed by direct observation in the kindergarten setting [11]. Small effects in vegetable intake are difficult to assess, therefore including high quality dietary methodology such as direct observation appears important. Parents of young children face a challenge when asked to report on child food intake away from home [13]. Hence, parents might not have been aware of the changes in their child’s vegetable intake in the kindergarten, as accuracy of parental report in such settings are questionable [13–15]. One may therefore speculate if the lack of long-term effects of the current intervention partly might be explained by the dietary assessment methodology used.

Further, a lack of long-term effects might also be a consequence of no follow-up on implementation in the second year of the intervention as the goal was to improve vegetable intake through changing the food environment and dietary practices in the kindergarten and the home. Moreover, it might be that parents viewed the components of the BRA-study as temporary activities and thus went back to previous vegetable routines after a while.

Strengths of the study were assessment of long-term effects of a multi-component intervention study with a sole focus on strategies to increase vegetable intake in an understudied age group. Moreover, by using mixed-models, all observations were included in the analyses, resulting in larger data sets compared with more standard approaches that only include complete cases. The mixed-model approach also ensured more correct estimation of standard errors and therefore more correct conclusions. At last, three measures of vegetable intake were explored and they showed slightly different intake patterns from baseline to follow-up 2 giving a broad picture of potential intervention effects.

### Conclusions

In conclusion, no significant long-term intervention effects in children’s intake of vegetables as measured by the parental reported frequency, variety or amount of vegetables were found. Further research to understand the best strategies to involve parents in dietary interventions studies is warranted and to also include high-quality dietary methodology in out of home settings.

## Limitations

The main limitation of the present study was the lack of objective measures for vegetable intake at follow-up 2 in out of home settings. Both our own research [11] and previous research [13–15] indicates that objective measures provide more accurate estimates of dietary intake in settings where parents may not be able to report properly on their child’s behalf. More than one person may have completed the questionnaires at the different time points for each child (e.g. mother/father), which may have impaired the consistency between measures. Besides, the questionnaires used have not been tested for reliability or validity. It is also likely that there was considerable variation in how the intervention was implemented.

## Data Availability

Data used and analyzed during the current study will be available from the corresponding author upon request, provided compliance with current legislation for application for data access in Norway.

## Abbreviations

The BRA-study: An acronym for the Norwegian words “Barnehage (kindergarten), “gRønnsaker (vegetables) and “fAmilie” (family)
Q1: questionnaire assessing vegetable frequency and variety
Q2: questionnaire assessing vegetable amount
